# Re-introducing non-optimal synonymous codons into codon-optimized constructs enhances soluble recovery of recombinant proteins from *Escherichia coli*

**DOI:** 10.1371/journal.pone.0215892

**Published:** 2019-04-23

**Authors:** Jennifer Konczal, Justin Bower, Christopher H. Gray

**Affiliations:** Drug Discovery Program, CRUK Beatson Institute, Glasgow, United Kingdom; Instituto Butantan, BRAZIL

## Abstract

Gene synthesis services have largely superseded traditional PCR methods for the generation of cDNAs destined for bacterial expression vectors. This, in turn, has increased the application of codon-optimized cDNAs where codons rarely used by *Escherchia coli* are replaced with common synonymous codons to accelerate translation of the target. A markedly accelerated rate of expression often results in a significant uplift in the levels of target protein but a substantial proportion of the enhanced yield can partition to the insoluble fraction rendering a significant portion of the gains unavailable for native purification. We have assessed several expression attenuation strategies for their utility in the manipulation of the soluble fraction towards higher levels of soluble target recovery from codon optimized systems. Using a set of human small GTPases as a case study, we compare the degeneration of the T7 promoter sequence, the use of alternative translational start codons and the manipulation of synonymous codon usage. Degeneration of both the T7 promoter and the translational start codon merely depressed overall expression and did not increase the percentage of product recovered in native purification of the soluble fraction. However, the selective introduction of rare non-optimal codons back into the codon-optimized sequence resulted in significantly elevated recovery of soluble targets. We propose that slowing the rate of extension during translation using a small number of rare codons allows more time for the co-translational folding of the nascent polypeptide. This increases the proportion of the target recovered in the soluble fraction by immobilized metal affinity chromatography (IMAC). Thus, a “de-optimization” of codon-optimized cDNAs, to attenuate or pause the translation process, may prove a useful strategy for improved recombinant protein production.

## Introduction

Widely available and affordable gene synthesis services have resulted in a decline in the use of PCR for the generation of recombinant expression clone inserts [1]. Along with this comes a ready opportunity to manipulate the nucleotide sequence of these open reading frames, removing codons that correspond to rare tRNAs in the expression host that may prove a bottleneck to protein expression [2]. The application of codon-optimized cDNAs is now prominent and often results in substantially improved output in protein production [3], and this trend is observed in our own *E*.*coli* expression laboratory. However, we consistently notice that a substantial proportion of the uplift achieved by codon-optimized cDNAs then partitions into the insoluble fraction, rendering it of poor quality and unavailable for native purification [4]. Troubleshooting this solubility issue using the classic optimisation variables (media type, *E*.*coli* strain, concentration of inducing reagent, temperature etc. [5, 6]) had no substantial effect in these systems. This led us to formulate a hypothesis that the markedly accelerated expression in codon-optimized systems somehow overwhelms the kinetics of protein folding meaning that much of the additional target generated partitions to inclusion bodies.

In order to attempt to shift this distribution back to the soluble fraction we determined to assess a number of alternative molecular methods that aim to attenuate the rate of expression of recombinant targets. As the use of classic optimisation strategies did not seem to improve output, perhaps imposing a more controlled speed of protein generation using molecular methods would enhance the folding of the nascent target.

We determined to assess the utility of three molecular approaches to enhance soluble recovery from codon-optimized systems expressing the recombinant human small GTPases KRas, RalA and Rac1. These efforts aim to manipulate either the rate of transcription, the rate of translation initiation or the rate of ribosome scanning and nascent polypeptide extension.

We altered the potency of the T7 promoter using mutations that had previously been shown to reduce expression rates in a fluorescent report assay [7], or reduced the direct interaction between the polymerase and promoter by atomic force microscopy [8]. Our results suggest that the degeneration of the T7 promoter sequence does reduce rates of expression but, in the systems we tested, the effect was severe and did not enhance the proportion of protein in the soluble fraction.

The overall rate of translation can be manipulated by reducing the efficiency of transcript engagement by the ribosome by employing weaker cognate start codons [9]. However, in our systems, the use of such alternative cognate start codons suppressed expression, but with no benefit to the soluble distribution.

Codon usage appears to have a critical effect on the efficiency of appropriate translation. The existence and distribution of synonymous codons in nature is now appreciated to be a non-random evolutionary strategy for modulating rates of protein translation [10]. Highly expressed genes are rich in common codons leading to a rapid expression and elevated accumulation [11]. Conversely, lower frequency synonymous codons are found in more moderately expressed genes prompting speculation that attenuation of transcript processing by the ribosome may play a part in regulating the levels of the corresponding protein [12]. This holds true in *E*.*coli*, where changes in efficiency of expression on introduction of alternative codons is well established [13]. Manipulation of normal tRNA populations in *E*.*coli*, supplementing with rare tRNAs, has been shown to induce an increase in aggregation of intrinsic proteins as a consequence of inappropriately accelerated translational extension [14]. Similarly, codon manipulation of translation rates has been shown to effect protein quality. For example, rapid *in vitro* expression of chloramphenicol acetyltransferase (CAT) from a codon optimized cDNA results in a decrease in protein activity [15].

We report the utility of manipulating synonymous codon usage in expression clones to drive an increase in soluble yield by tuning down the translation extension rate of target proteins. Reintroducing a small number of rare non-optimal codons into the cDNA of each expression plasmid resulted in progressive gains in soluble recovery, despite a reduction in total expression levels. We propose that introducing a limited number of rare codons into otherwise optimised cDNAs slows or pauses nascent polypeptide extension by the ribosome sufficiently to allowing a greater proportion of protein to adopt the correct soluble conformation during co-translational folding. The net result is a substantial increase in soluble recovery following native purification.

## Materials and methods

### Creation of a panel of modified GTPase expression vectors

Various alternative cDNAs were generated that encode the G-domains of the human small GTPases KRas (1–169), RalA (8–183) and Rac1 (1–184). Initially both the native human sequences and fully codon-optimized versions were generated by gene synthesis (Genewiz). These cDNAs were cloned to the NcoI and XhoI sites of pBDDP-SPR3 which allows expression with an N-terminal double-His_8_ affinity tag that includes two repeat octa-histidine motifs separated by a flexible 28-mer linker [5, 16]. A series of mutations were introduced to the codon-optimized clones, either by standard site directed mutagenesis or using further gene synthesis. This resulted in a panel of expression vectors for GTPase production that differed either in the sequence of the T7 promoter, the nature of the translational start codon or by the presence of selected rare codons in the otherwise optimized sequence.

### Expression of GTPases

Plasmids were transformed into the BL21 (DE3) pLysS strain of *E*.*coli* (Merck Millipore) or Rosetta2 DE3 pLysS (Merck) and the transformed cells were cultured overnight at 37°C in Luria Bertani broth (Sigma Aldrich) supplemented with 30μg/ml kanamycin sulfate (Melford Laboratories Ltd). A volume of 20ml of this overnight starter culture was used to inoculate each of 1 litre of Terrific Broth (Sigma Aldrich) broth supplemented with 30μg/ml kanamycin sulfate. When the OD_600_ reached approximately 0.8, protein expression was induced by the addition of IPTG (0.1mM, 0.5mM or 1mM) (Melford Laboratories Ltd) and expression was allowed to proceed either for 16 hours at 18°C, or 4 hours at 37°C. A volume of 40ml was sampled from the 1 litre cultures and cells were harvested by centrifugation at 3220xg for 15 minutes (Eppendorf 5810R centrifuage and A-4-62 rotor). The supernatant was discarded and the cell pellets were snap frozen in liquid nitrogen prior to storage at -80°C.

### SDS-PAGE analysis of GTPase expression

To analyse expression of the recombinant target in the total cell protein (TCP) a 1ml sample of the end point expression culture was collected and the OD_600_ measured. The sample was centrifuged at 7500xg for 2 minutes in a microfuge (Eppendorf 5415R and F45-25-11 rotor) and the supernatant discarded. The cell pellet was resuspended in 200μl of 50mM Tris HCl pH7.5, 500mM NaCl and 2μl Benzonase. In order to ensure a well resolved and appropriately loaded SDS-PAGE analysis, an appropriate and consistent concentration of TCP was loaded in each sample. The total protein content of each SDS-PAGE gel sample was standardised using a simple calculation where (17/OD_600_)μl of cell suspension was added to 10μl SDS-PAGE loading dye (Invitrogen). Samples were heated for 4 mins at 95°C and analysed on 4–12% Bis-Tris NuPAGE gel (Invitrogen).

### Denaturing purification of GTPases to quantify total expression levels

Frozen cell pellets (from 40ml culture), resuspended in 800μl of 50mM Tris HCl pH7.5, 750mM NaCl, 8M urea, were recovered from -80°C storage and allowed to thaw to room temperature. Lysis was completed on three successive freeze-thaws between dry ice and 37°C and the material was allowed to solubilise for 24 hours at 4°C with end over end mixing. The lysate was clarified by centrifugation at 16,100xg for 10 minutes (Eppendorf 5415R and F45-25-11 rotor) and the supernatant collected for isolation of solubilized protein. Purification of His-tagged protein was achieved using 100μl of high density cobalt resin (ABT) immobilized in small scale Proteus spin columns (Generon). Immobilized metal affinity chromatography (IMAC) columns were equilibrated with 5 washes with binding buffer (50mM Tris HCl pH7.5, 750mM NaCl, 6M urea, 10mM Imidazole) using microfuge centrifugation at 200xg for 30 seconds to process. To ensure the loading did not overwhelm the column, 100μl of solubilised material (thus derived from 5ml of original expression culture) was added and allowed to incubate at room temp for 10 mins with the resuspended cobalt resin to allow binding. The IMAC beads were then collected by centrifugation which was followed by 5 x 500μl washes with wash buffer (50mM Tris HCl pH7.5, 750mM NaCl, 6M urea, 25mM Imidazole). Finally, the purified protein was eluted by resuspending the IMAC beads in 70μl elution buffer (50mM Tris HCl pH7.5, 750mM NaCl, 6M urea, 500mM Imidazole), incubating for 5 minutes at room temperature and recovering the eluate by centrifugation. Purified material was characterised by running 5μl of eluate on SDS-PAGE, and quantified as a milligram per litre yield using a standard Bradford assay. Samples of sample flow-through and wash flow-through were collected for SDS-PAGE analysis to indicate that there was no un-captured material during the process to ensure accurate quantitation of yields.

### Native IMAC purification of GTPases to quantify soluble expression yields

Frozen cell pellets (from 40ml culture) were recovered from -80°C storage and allowed to thaw to room temperature. Pellets were resuspended in 300μl of a mixture of 50% binding buffer (*20mM Tris HCl pH7*.*5*, *500mM NaCl*, *10mM Imidazole*), 50% bugbuster reagent (Millipore) and 2μl Benzonase nuclease. Lysis was completed on three successive freeze-thaw between dry ice and 37°C. The lysate was clarified by centrifugation at 16,100xg for 10 minutes and the supernatant collected. Purification of His-tagged protein was achieved using 100μl of high density cobalt resin (ABT) immobilized in small scale Proteus spin columns (Generon). IMAC columns were equilibrated with 5 washes with binding buffer (20mM Tris HCl pH7.5, 500mM NaCl, 10mM Imidazole) using microfuge centrifugation at 200xg for 30 seconds to process. Clarified lysate was added and allowed to incubate at room temperature for 10 mins with the resuspended cobalt resin to allow binding. The IMAC beads were then collected by centrifugation which was followed by 5 x 500μl washes with wash buffer (20mM Tris HCl pH7.5, 500mM NaCl, 25mM Imidazole). Finally, the purified protein was eluted by resuspending the IMAC beads in 70μl elution buffer 20mM Tris HCl pH7.5, 500mM NaCl, 300mM Imidazole, incubating for 5 minutes at room temperature and recovering the eluate by centrifugation. Purified material was characterised by running 10μl of eluate on SDS-PAGE, and quantified as a milligram per litre yield by absorbance at 280nm using a nanodrop spectrophotometer and the appropriate molecular extinction co-efficient for each protein. Samples of lysate flow-through and wash flow-through were collected for SDS-PAGE analysis to indicate that there was no un-captured material during the process to ensure accurate quantitation of yields.

## Results

### Evaluation of the uplift in expression and purification recovery of small GTPases on codon optimisation

**Fig 1:**
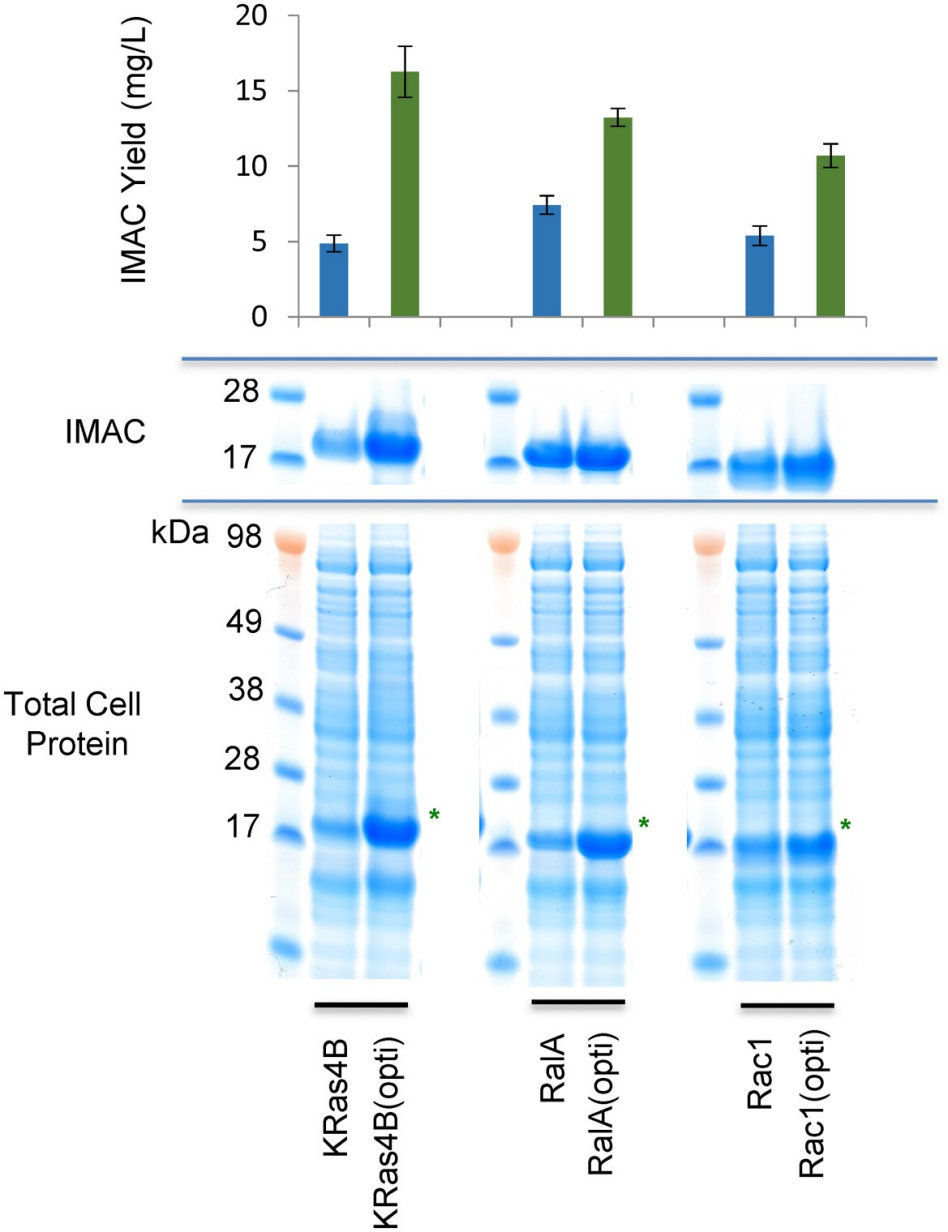
Comparison of yields from native and codon optimized sequences. SDS-PAGE analysis and quantification of soluble fraction IMAC yield for GTPase expression clones containing the *Homo sapiens* non-optimized sequence cDNA or a fully codon optimised version. The position of the target GTPase is indicated by a green asterisk. All IMAC purifications were performed as n = 3 technical replicates, deriving from distinct replicate 1L cultures.

A set of clones designed to express the G-domains for three human small GTPases, KRas (1–169), RalA (8–183) and Rac1 (1–184) were produced by gene synthesis and cloning. Options for expression from both the native *H*. *sapiens* sequence or a fully codon optimised cDNA were established. In each case, the levels of both total expression and soluble purified target were established on expression in the BL21DE3 pLysS strain of *E*.*coli* (Fig 1). Consistent with many previous examples, codon optimisation substantially increased expression and markedly enhanced cytoplasmic levels of each target GTPase was observed in the total cell protein analysis. An approximate 5-fold enhancement is observed in comparison to the native *H*. *sapiens* cDNAs; total expression yields for codon optimised KRas is 61.05mg/litre compared to 11.85mg/L for the native sequence (Table 1). However, a 5-fold increase did not translate to the recovery of soluble target from IMAC purification, where a more modest 2–3 fold uplift was observed. Frustratingly, a substantial proportion of the additional target obtained through elevated expression partitions to the insoluble fraction and is thus unavailable for recovery by native purification techniques. Quantification of the performance of the KRas expression systems demonstrates that whilst the more poorly expressing native sequence clone results in the recovery of 41.1% of the total material on IMAC, the codon optimized option sees only 26.64% returned from purification of the soluble fraction. Thus this tendency for accelerated expression to correlate with reduced solubility means that much of the performance benefit of the codon optimized clones was lost to the insoluble compartment.

**Table 1:** Detailed quantification of expression and soluble fraction recovery of a panel of KRas 1–169 expression clones.

Protein	Total (mg/L)	Soluble (mg/L)	% soluble
KRas4B 1–169 (hs)	11.8	4.9	41.1
KRas4B 1–169 (Codon optimised)	61.0	16.3	26.6
KRas4B 1–169 (opti) 0.1mM IPTG	54.7	13.8	25.3
KRas4B 1–169 (opti) 0.5mM IPTG	61.0	16.3	26.6
KRas4B 1–169 (opti) 1.0mM IPTG	65.2	13.0	19.9
KRas4B 1–169 (opti) Rare I46	47.0	14.9	31.6
KRas4B 1–169 (opti) Rare I84	53.2	19.4	36.5
KRas4B 1–169 (opti) Rare R164	53.4	22.2	41.7
KRas4B 1–169 (opti) Rare I84/R164	50.7	34.9	68.8
KRas4B 1–169 (opti) Rare I46/I84/R123/R164	50.2	41.6	82.8

**Fig 2:**
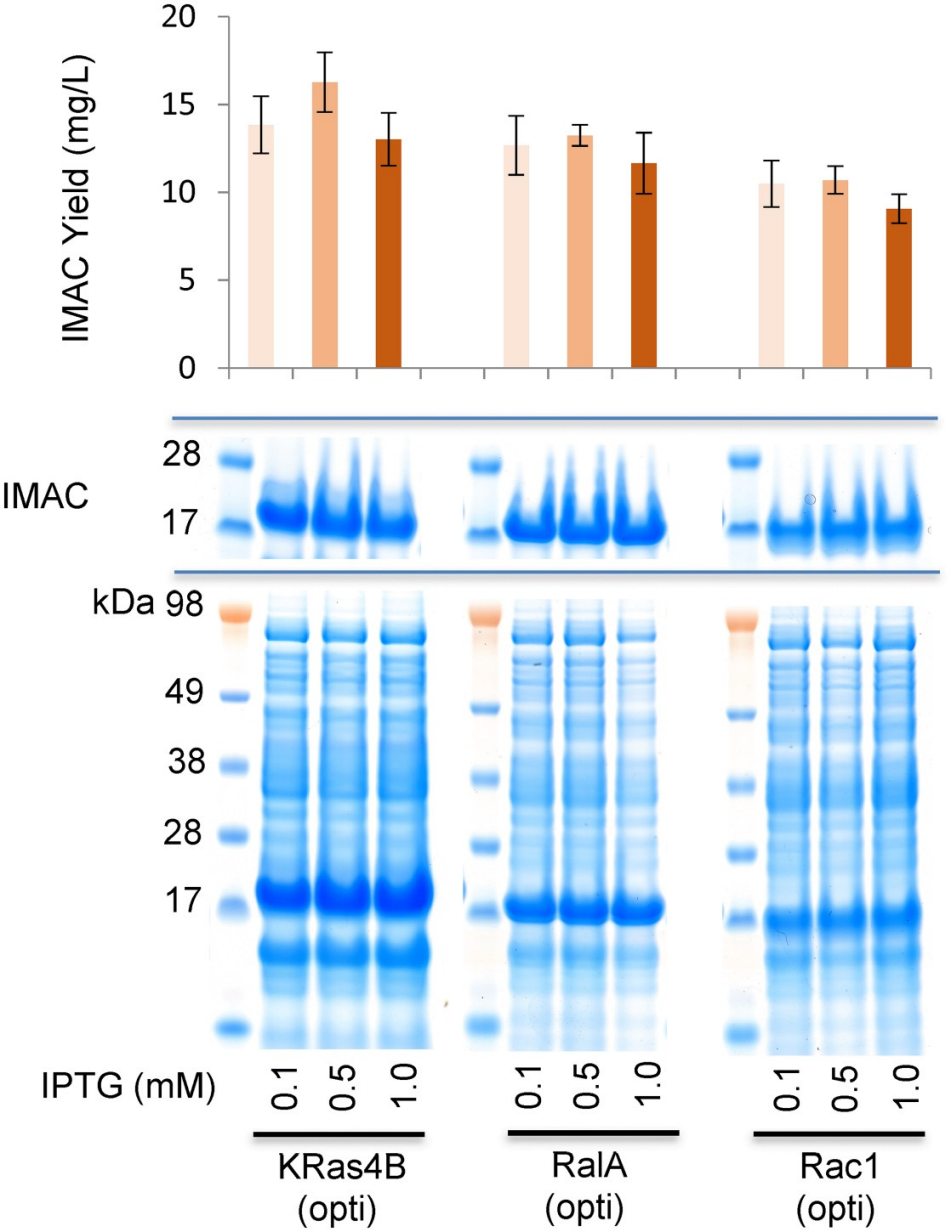
Tuning induction for soluble expression. SDS-PAGE and soluble fraction IMAC yield of codon optimized GTPase expression clones on titration of IPTG at induction. For each example, IPTG was applied at 0.1, 0.5mM or 1mM concentrations.

Several traditional approaches to further optimisation of solubility were attempted, including the titration of IPTG concentrations on induction of each expression system in an attempt to tune down the levels of transcription of each target. However, these did not provide any significant benefit. Indeed, in the case of each GTPase, variation of IPTG concentration from 0.1mM to 1mM showed little difference in the performance of each codon optimized clone with equivalent levels of expression and soluble recovery occurring in each case (Fig 2) with total yields little changed and the soluble recovery remaining between ca. 20–25% (Table 1). It would appear that the activity of the T7 promoter in these clones is binary when induced using typically employed IPTG concentrations.

The uplift in expression on codon optimization is noted along with the concurrent drop in the percentage of soluble material. IPTG titration, as an attempt to tune transcription, had no significant effect on expression levels of solubility. However, the progressive introduction of selected rare codons back into the optimized open reading frame resulted in a progressive improvement in soluble yields despite a concomitant reduction in the total expression levels.

### Protein expression and solubility on degeneration of the T7 promoter

**Fig 3:**
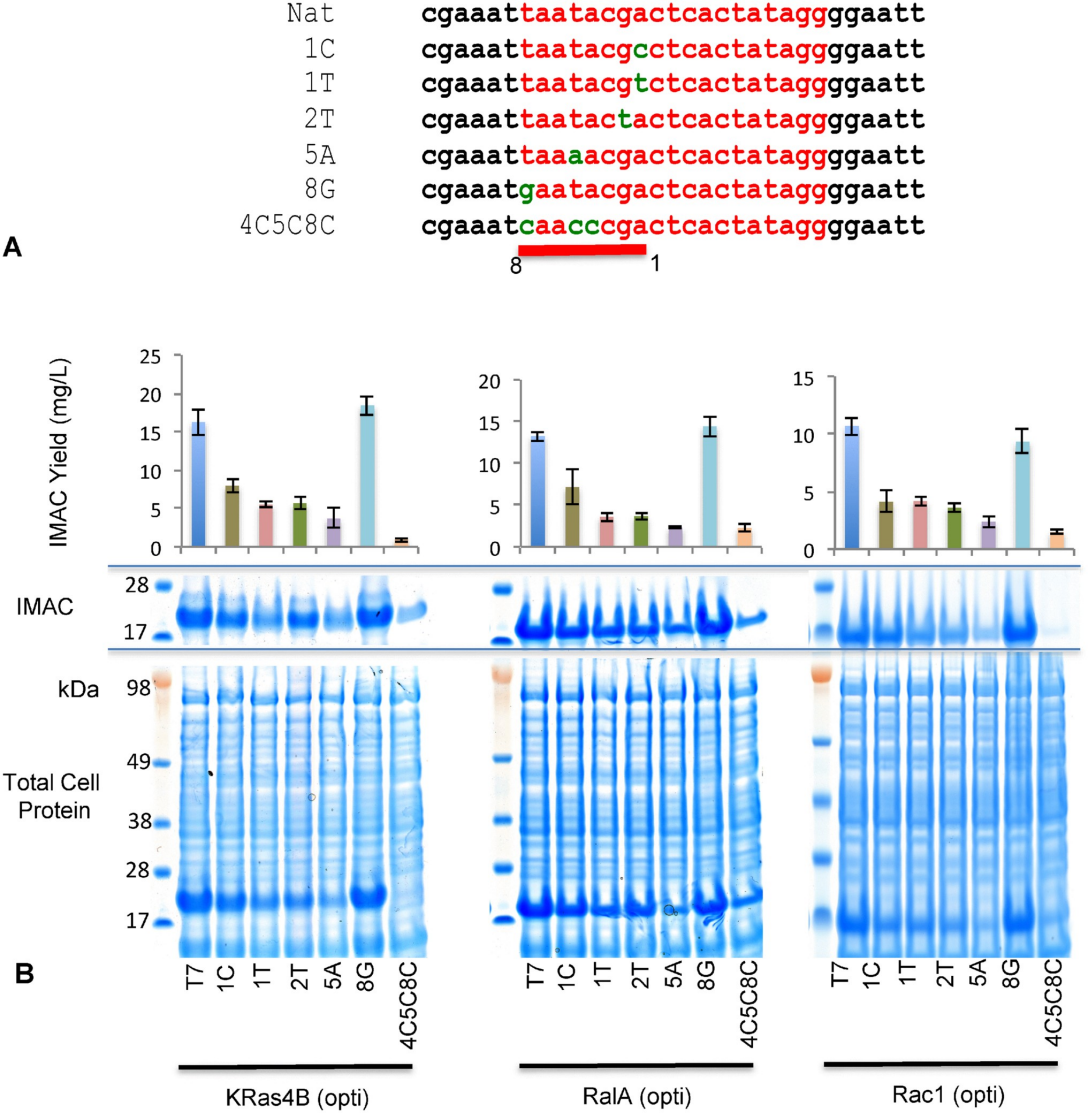
Solubility is not improved by altering the T7 promoter. (A) A series of attenuating point mutations, indicated from the literature, were established in the T7 promoter region of each pBDDP-SPR3 expression clone. (B) SDS-PAGE and soluble fraction IMAC output of codon optimized GTPase expression clones with degenerated the T7 promoters.

A panel of mutant T7 promoter variants were generated, to attenuate transcription to varying degrees by reducing the efficiency of interaction with the T7 polymerase. In total, 6 variant promoters were generated for each of the GTPase systems (Fig 3A) and assessed for expression and soluble yield. Results were consistent across all GTPases with the majority of promoter variants resulting in a severe reduction in both total and soluble yield (Fig 3B). Only 1 variant, T7 8G, appear to retain or marginally enhance expression. Importantly, analysis of the soluble recovery tracked accurately with the levels of total expressed protein indicating that degeneration of the T7 promoter, and the reduction in transcription, had no substantial effect on the proportion of product that partitioned into the soluble fraction (Table 2). In instances where a degree of improved solubility was observed, the depressed levels of expression eliminated any benefit as yields were problematically low.

**Table 2:** Milligram per litre yields and soluble partitioning from KRas expressions from different T7 promoter variants.

Protein	Total (mg/L)	Soluble (mg/L)	% soluble
**KRas4B 1–169** **(native T7)**	61.0	16.3	26.6
**KRas4B 1–169** **Pro1C**	37.7	7.9	21.0
**KRas4B 1–169** **Pro1T**	17.0	5.5	32.3
**KRas4B 1–169** **Pro2T**	20.4	5.6	27.8
**KRas4B 1–169** **Pro5A**	14.8	3.0	20.6
**KRas4B 1–169** **Pro8G**	59.5	18.4	31.0
**KRas4B 1–169** **Pro4C5C8C**	5.4	0.9	17.6

### Protein expression and solubility on variation of the translational start codon

**Fig 4:**
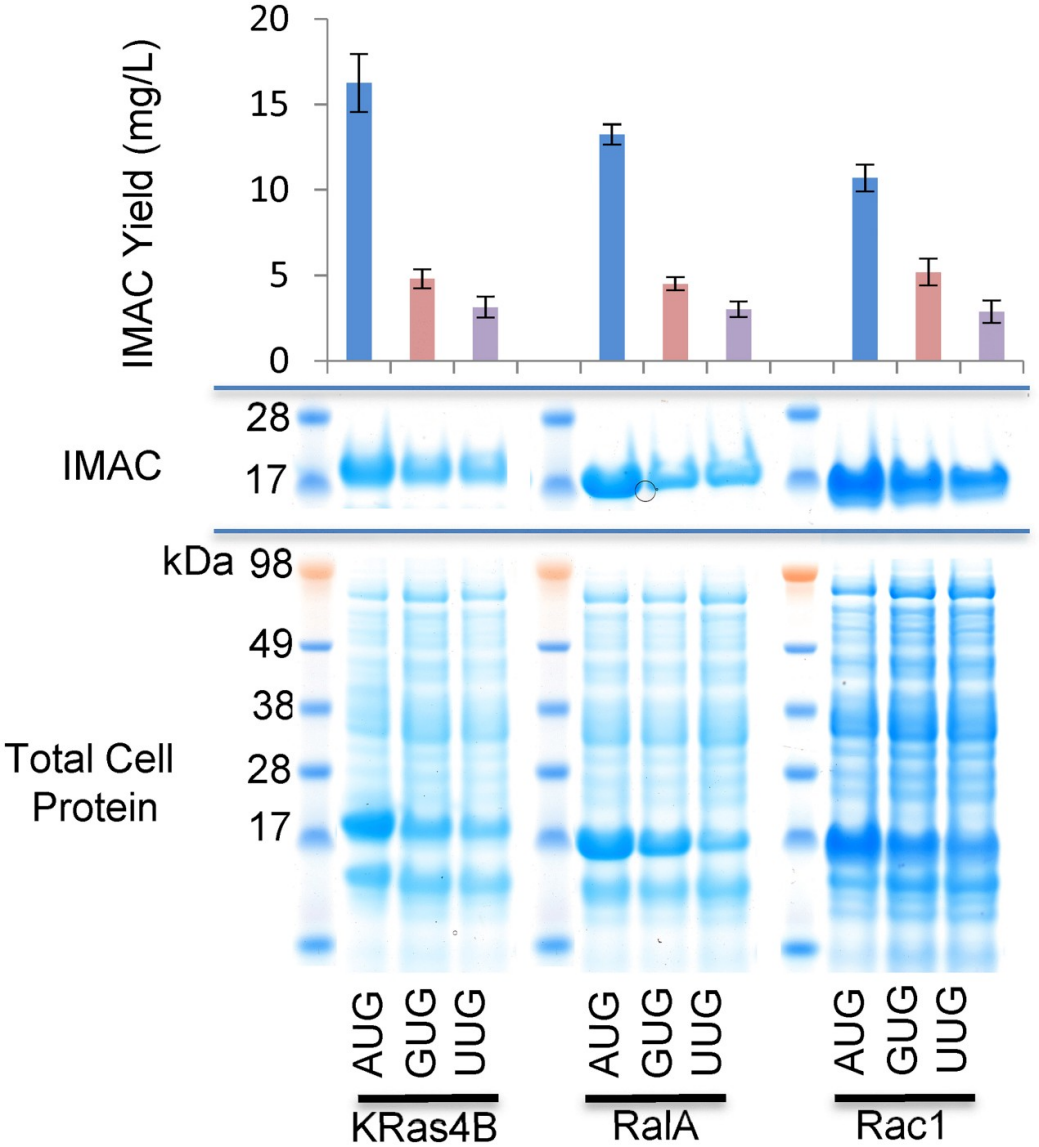
Solubility is not improved by altering the translational start codon. (A) SDS-PAGE and soluble fraction IMAC yield of codon optimized GTPase expression clones where the translational start codon was altered from the normal AUG to either GUG or UUG.

As efforts to improve soluble recovery by reduction the rate of translation proved ineffective, we assessed the utility of reducing the rate of initiation of translation by replacing the AUG start codon with different cognate alternatives. AUG is predominantly the most favoured start codon in nature, and replacing this with most other codons results in a catastrophic reduction in translation of transcripts. However, a moderate level of translation is retained by both GUG and UUG as initiating codons [17]. Expression and solubility profiles for each GTPase, using AUG, GUG and UUG as the start codon, revealed that modulation of translation initiation was also largely ineffective in improving soluble recovery (Fig 4). As in the case of the majority of degenerated T7 promoters, the use of alternative start codons had no effect on solubility, 26.64–27.18% for KRas variants for example (Table 3), and reduced the overall expression levels significantly.

**Table 3:** Milligram per litre yields and soluble partitioning from KRas expressions from different cognate start codons.

Protein	Total (mg/L)	Soluble (mg/L)	% soluble
**KRas4B 1–169** **(AUG)**	61.0	16.3	26.6
**KRas4B 1–169** **(GUG)**	17.7	4.8	27.2
**KRas4B 1–169** **(UUG)**	11.6	3.1	27.0

### Protein expression and solubility on introduction of selected rare codons into the codon optimised cDNA

As modulation of both transcription and translation initiation were of no benefit, we extended our experiments to address approaches which aimed to alter expression performance by reducing the rate of translation via the slowing or pausing of the otherwise efficient scanning of recombinant mRNA transcripts by the ribosome. In each case, 4 amino acids positions were selected at various positions in the sequence of each GTPase (being either an Arg, Ile or Leu) that are encoded by synonymous codons in the *H*. *sapiens* sequence which are rare in *E*.*coli*. We established a panel of GTPase expression constructs where either single or multiple codons for these residues were substituted with non-optimal codons which are known to be of low abundance in *E*.*coli* (Arg;CGG, Ile; AUA, Leu;CUA) (Table 4).

**Table 4:** Positions selected for substitution with non-optimal codons.

	Single	Double	Multi
**KRas4B 1–169**			
I46	Y		Y
I84	Y	Y	Y
R123			Y
R164	Y	Y	Y
**RalA 8–183**			
L32	Y		Y
R108	Y	Y	Y
R176			Y
R178	Y	Y	Y
**Rac1 1–184**			
R66	Y		Y
R102	Y	Y	Y
L143			Y
R174	Y	Y	Y

Array of constructs introducing single or multiple rare codons to the optimised expression clones for each GTPase (R: CGG, I: AUA, L: CUA). Positions were chosen arbitrarily to span relatively regular intervals throughout each open reading frame.

**Fig 5:**
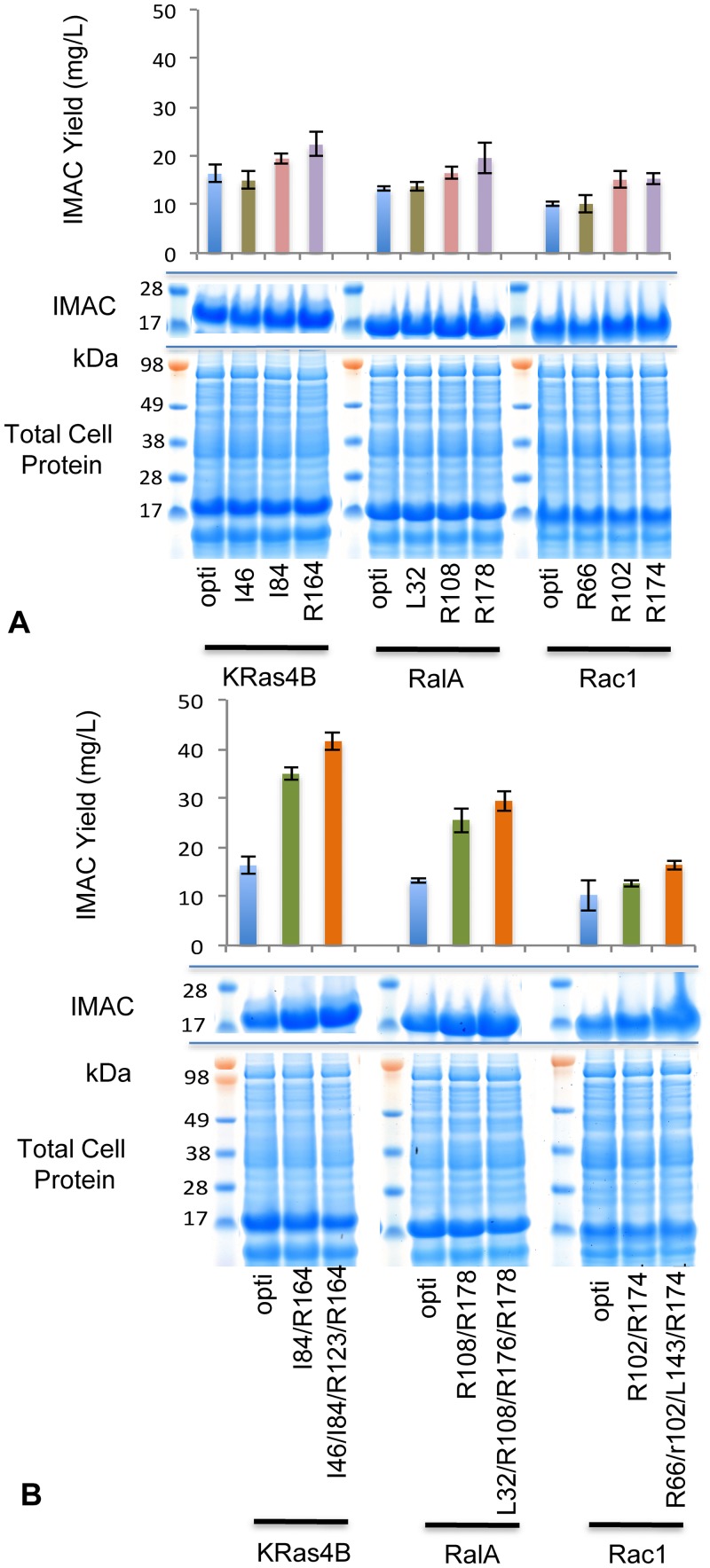
Introducing non-optimal synonymous codons increases soluble recovery. (A) Expression output from a series clones containing single rare codons positioned progressively through the otherwise codon optimised (opti) open reading frame. (B) Improvements in soluble fraction IMAC yield and soluble partitioning from expression constructs with either 2 or 4 rare codons imposed on the optimised open reading frame.

The introduction of a single rare codon at any position in the cDNA had marginally negative effects on total expression levels with only slightly depressed amplitudes of target expression analysis of all cultures (Fig 5A, Table 1). However, returns from IMAC purification did improve in most cases with an improvement in yield of up to 37.7% observed in the best responding clone (KRas R164). The trend suggests that the greatest improvement in soluble yield is expected from rare codons introduced towards the 3’ end of the transcript. Most notably, the increased recovery from IMAC was realized as a consequence of improved solubility. Despite the fact that each rare codon resulted in a slight decrease in the total levels of expression, the proportion of target partitioning to the soluble fraction was consistently elevated (solubility for codon optimized KRas; 26.64%, KRas R164; 41.68%).

Combining rare codons had a more marked effect. In these cases the reduction in total expression levels were clear, particularly in the KRas and RalA systems (Fig 5B). The greatest margin of uplift in IMAC yields was observed on the introduction of 2 rare codons to each cDNA, with a greater than 100% improvement in both KRas and RalA production, with a more modest response in the Rac1 system. In comparison to the optimized system, KRas IMAC yield improved by 114% (34.92mg/L versus 16.27mg/L), as consequence of a shift in solubility from 26.64% of total expressed target to 68.82% (Table 1). Interestingly, the degree of solubility observed from the de-optimized vectors was substantially higher than the solubility observed on production from vectors containing the native *H*.*sapiens* cDNA (KRas; 68.82% versus 41.1%).

Further gains in soluble recovery were observed on incorporation of 4 rare codons positioned throughout the transcript (Fig 5B, Table 1). In the cases of KRas and RalA, it would appear that the greatest change in performance was achieved from the 2 codon substitutions with only a smaller further enhancement when the rare codon count increased to 4 (KRas production further improved by 6.64mg/L and a 14% additional lift in solubility). However, IMAC output suggests that the Rac1 system benefitted particularly from the 4 rare codons.

### Supplementing with rare tRNAs abolishes the uplift conferred by de-optimized sequences

**Fig 6:**
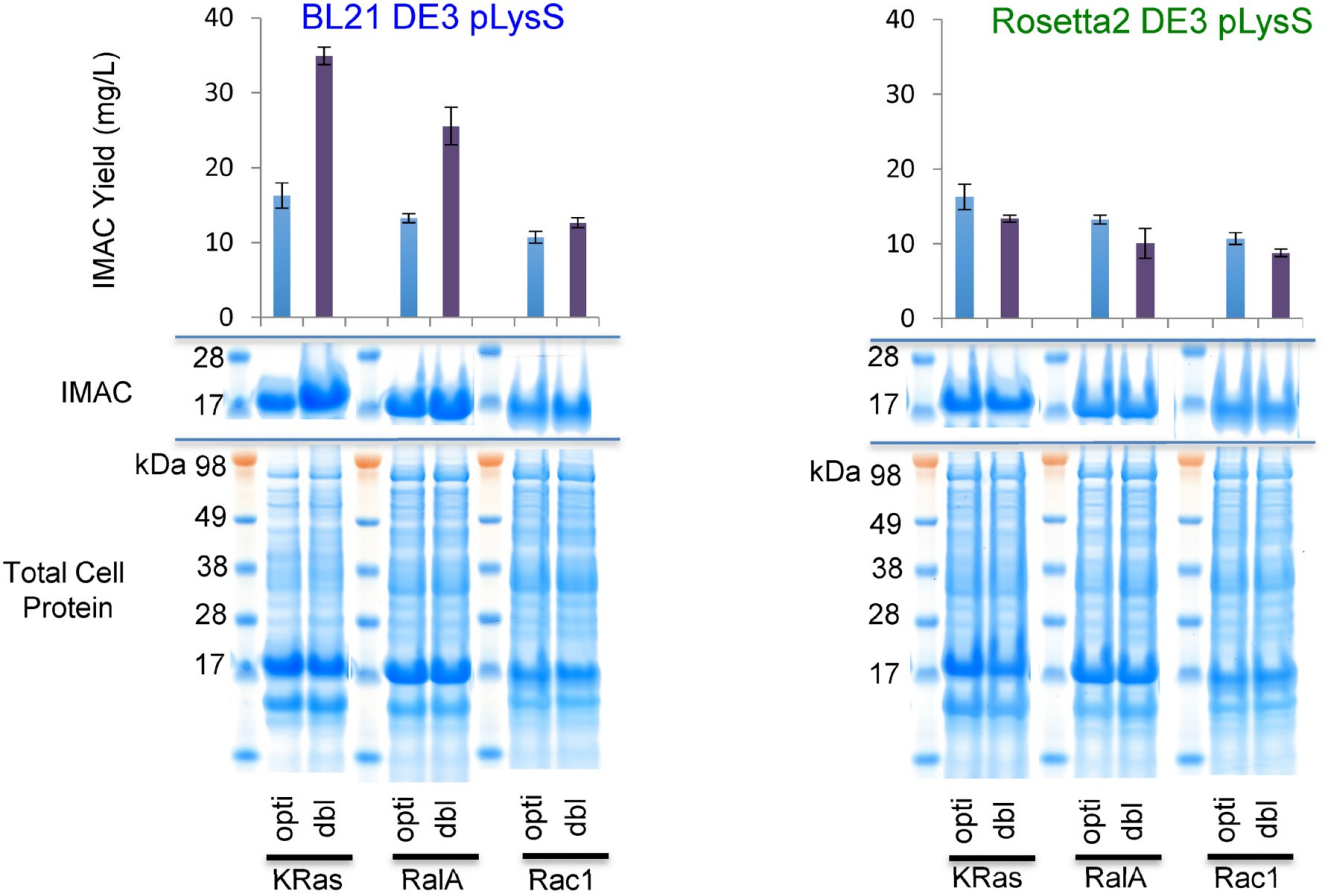
The benefit of non-optimal codons is lost on supplementation with rare tRNAs. The improvements observed on the introduction of two rare codons (dbl) into each optimized reading frame is dependent on the relative lack of the corresponding tRNA. Whilst the uplift is sustained in a BL21 DE3 pLysS strain, the benefit of rare codons is lost in Rosetta2 DE3 pLysS where the minority tRNAs corresponding to modifications are supplemented from an accessory plasmid.

In order to confirm that the improvements observed were indeed due to the presence of rare codons, we repeated expression of each protein from the codon optimized (opti) and 2-codon de-optimised (dbl) expression vectors in Rosetta2 DE3 pLysS cells. Rosetta2 strains contain a pRARE2 plasmid that supplements the cell with the rare tRNAs that correspond to the non-optimal codons in the modified cDNAs. Consequently, the presence of these additional tRNAs restored the performance of the de-optimized clones to that observed in the fully codon-optimized systems (Fig 6).

**Fig 7:**
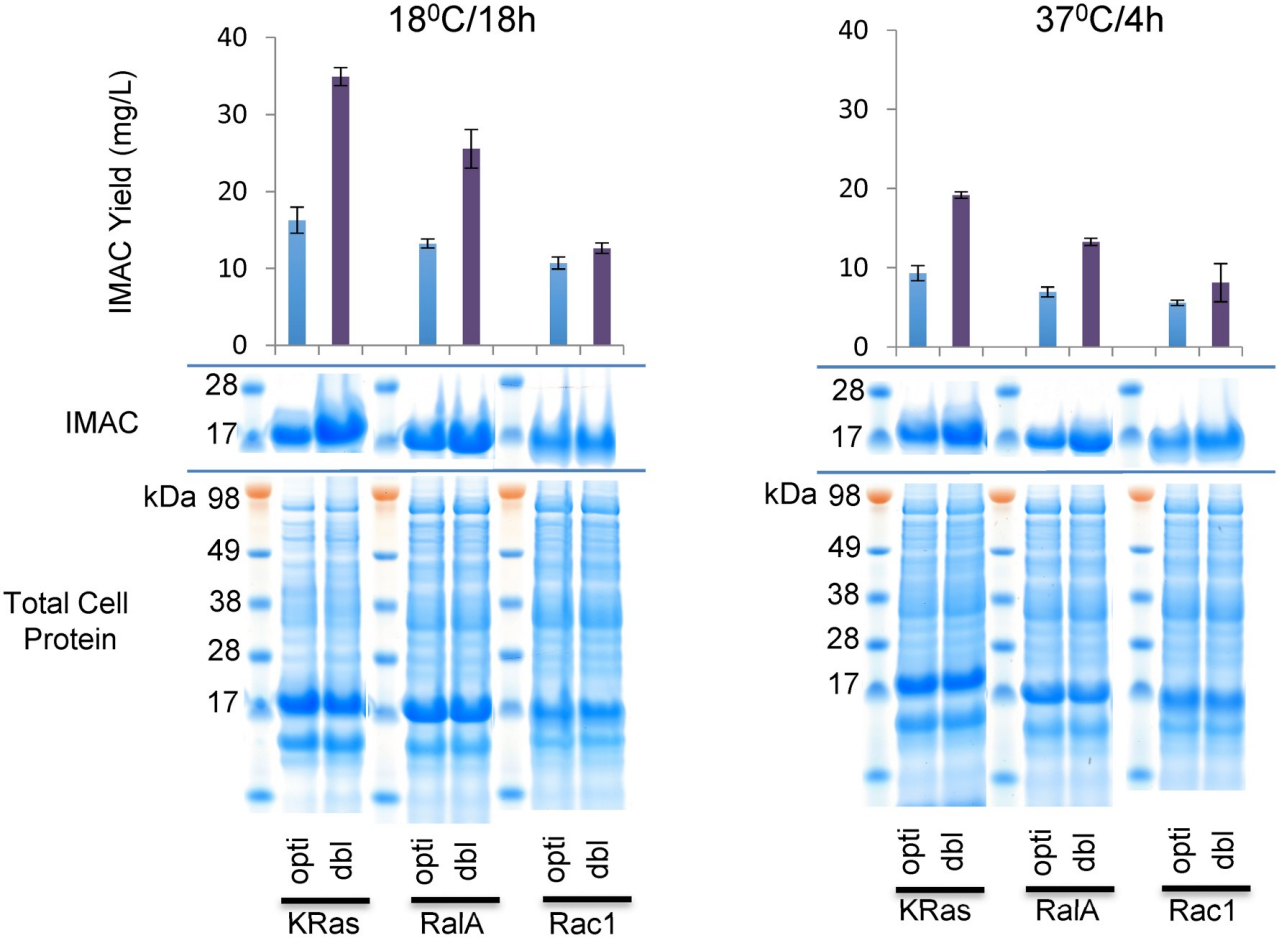
Improved soluble yield offers options for workflow efficiency. A comparison between the performances of clones demonstrates that the yield from the dbl modified clone following a short 4 hour expression at 37°C is roughly equivalent to the output from the fully optimised clone following an overnight 18°C expression batch. Thus the improved performance offers the opportunity to compress expression times if throughput rather than yield is the most important factor.

The improved solubility realized by the de-optimized systems can confer operational advantage beyond simple protein recovery. We noticed that the IMAC yield from the de-optimized systems, grown quickly at 37°C for 4 hours, matched the yield from the originally fully optimised clones after at 18°C/18 hour expression culture (Fig 7). Consequently, if throughput in the culturing facilities is the priority rather than yield, these improved systems offer the opportunity to compress expression culture runs freeing up incubator capacity for operational benefit.

## Discussion

The use of codon-optimized reading frames aims to eliminate any translational bottleneck imposed by a shortage of rare tRNAs as a consequence of the differing codon usage profile between the originating species of the target protein and that of the expression host cell [2]. As a result, the synthetic mRNA transcript is scanned and processed by the ribosome very efficiently, maximising the extension rate of the nascent polypeptide [12]. However, this accelerated expression rate can be too potent, resulting in a substantial amount of the increased yield partitioning to the insoluble fraction. Often, the translation rate of these optimized transcripts overwhelms the co-translational folding kinetics of the target protein [12]. As a result, the increased gain from codon-optimized cDNAs is somewhat frustrated.

As classic optimization approaches had minimal effect on our model GTPase expression systems, we evaluated alternative molecular approaches to modulate expression rates, with the hope of maximising soluble recovery of recombinant protein. A typical first recommendation in tackling solubility problems is to tune down the translation rate to reduce the concentration of recombinant transcript in the system. There are some specialist vectors with sugar analogue-tunable promoters that claim to be ideal for this approach [18, 19], but they remain in occasional use despite years of availability. Rather, the lac repressed T7 polymerase/T7 promoter remains the system of choice for most bacterial protein expression laboratories even though it cannot be tuned to any great degree [20]. We observed that titration of IPTG, to tune induction of transcription from the T7 promoter, was ineffective in altering both total and soluble yield. Indeed, titration of IPTG in our test systems confirmed our consistent observation that the T7 promoter is often a binary switch and, once activated, will transcribe at maximal levels with no respect to the inducing IPTG tension.

There are substantial number of studies that report the effects of point mutations on T7 promoter activity (recently, [21, 22]). We introduced a series of mutations to the T7 promoter of our pBDDP-SPR3 expression systems [16] that had previously been shown by others to reduce the transcription rates. Although these alterations do not provide a dynamic tuneable promoter, they do allow us to examine the effect of reduced transcription on the soluble partitioning of our GTPases. In all but one modification, the expression was substantially reduced. However, disappointingly, the recovery of soluble GTPase reduced in a co-linear manner demonstrating that transcript abundance had no real effect on soluble portioning for these proteins.

In a recent study, Hecht *et al* assessed the capacity for translation initiation by all 64 codon triplets [17]. Measurement of the expression of GFP and nanoluciferase reporters indicated that a number of alternative start codons can initiate translation, but only the typical AUG or the near cognates GUG or UGG resulted in any substantial expression, where output >10% of the AUG is retained. Reducing the efficiency of translational initiation, by using weaker start codons, had a similar result, reducing expression rates to a poor level, but having no effect on the percentage of recombinant target available for native purification.

As both transcript abundance or translation initiation did not seem to control solubility or folding, we assessed the strategy of attenuating the rate of transcript scanning by the ribosome, effectively slowing the extension of the nascent polypeptide. The persistence of rare codons in nature alludes to an evolved mechanism, whereby variation in the dynamics of translation are used to regulate protein abundance and function [23]. Mimicking this mechanism in protein production biotechnology, re-introducing a small number of rare codons back into codon-optimized cDNAs, proved effective in our small GTPase expression systems. Unsurprisingly, reducing the processing rate of the ribosome did exert a downward pressure on our total protein yields. However, the co-incident increase in the solubility of the target more than compensated for this and the net yields from IMAC purification were substantially improved.

Each GTPase expression system responded to a different degree and it is probable that the optimum number and positioning of rare codons will differ for each target. With the limited dataset presented herein, we are long way from establishing a predictive solubility algorithm for construct design. Such a tool will require a better knowledge of the fundamentals of co-translational folding as well as a broader and more comprehensive set of observations. Improved tools are emerging that allow enhanced codon usage analysis as well as a theoretical prediction of the impact this may have on the efficiency of co-translational folding [24]. However, designing constructs to manipulate co-translational folding for solubility may remain an empirical task for some time.

One consistent trend presents itself in the soluble recovery data for the single rare codon substitutions. Consistently, the benefit afforded by a single rare codon was most pronounced when the substitution was positioned towards the 3’ end of the transcript. This would impose the slowing or pausing event when the C-terminal end of the nascent protein was translating. As a consequence, the largest portion of the new protein would have emerged from the ribosome and be given more time to adopt the correct soluble fold prior to translational termination. Indeed, this strategy seems to be true in nature as a number of genes show a clustering or rare codons at the 3’ terminus of the open reading frame [23], presumably allowing the nascent product to linger on the ribosome just before translation is terminated.

As understanding increases, further benefit may be gained by a more detailed design strategy. For instance, greater benefit may be realised by positioning rare codons to impose pausing during the translation of linker regions between the distinct domains of multi-domain targets. Even with single domain proteins, it may be of benefit to schedule the pauses to coincide with the emerging of loop regions from the ribosome channel, ensuring individual secondary structure elements can emerge without interruption.

## Conclusions

We had previously noted that the expression kinetics from our codon-optimized vectors often seemed “too hot”, pushing the gains into the insoluble fraction. However, conventional optimisation methods did not assist. These data suggest that the reason for the solubility issues did not reside in over production of transcript or an over-frequent engagement with the ribosome to initiate translation. Rather, the fully optimized transcript was scanned by the ribosome too efficiently, unfettered by any restriction in the tRNA population. As a consequence the extension rate of the nascent polypeptide outpaced the kinetics of co-translational folding resulting in a frustrating increase in insoluble material. By re-introducing a small number of rare codons back into the optimised cDNA, the translation rate is somewhat slowed, allowing more productive co-translational folding and substantially increasing soluble yields recovered by native IMAC purifications.
